# Towards automated metabolome assembly: application of text mining to correlate small molecules, targets and tissues

**DOI:** 10.1186/1758-2946-3-S1-P19

**Published:** 2011-04-19

**Authors:** P Moreno, KV Jayaseelan, C Steinbeck

**Affiliations:** 1 Cheminformatics and Metabolism Group, European Bioinformatics Institute (EBI), Cambridge, CB10 1SD, UK

## 

How many species are there on Earth? Single celled organisms aside a few million are known. How many species had their genomes sequenced so far? Around two thousand. Approaching the era of a new genome sequence per week, it is fair to wonder: How many metabolomes have been compiled? The metabolome could be considered the ultimate phenotypic expression of the cell, and yet, we barely have one [1]. The metabolome refers to the complete set of small molecules (< 1500Da) present on a biological sample or organism [2]. Among all omics, metabolomics is probably the most unique to each individual in terms of the variation of its elements. Here we define Automated Metabolome Assembly (AMA) as a set of techniques to predict the metabolome of an organism based on the complete set of boundary information available (gene sequence, proteomics, bibliomics, etc.). As a first step towards Automated Metabolome Assembly we report the implementation of a text mining resource based on the existing EBI text mining infrastructure [3] to address the problem of finding co-occurrences of chemical entities, proteins, organisms, and tissues/cell types terms. We created a workflow based on a database holding 365 million of relations between these terms (proteins, metabolites, organisms and tissues) and PubMed citations, obtained from the whole PubMed collection up to September 2009. Dictionaries of terms for each kind of entity were generated from different ontologies. All known metabolites present in liver were obtained from the latest version of HMDB. The text mining results were compared to this reference set. Close to 90% of the reference set shows co-occurence of our liver-related tissue ontology terms with the respective metabolite names, demonstrating that this text-mining workflow can form an important building block for a comprehensive system for metabolome prediction.
